# Comparative Effect of African Walnut Oil and Docosahexaenoic Acid on Hematological and Oxidative Stress Markers of Female Wistar Rats With 7,12‐Dimethylbenz[a]anthracene‐Induced Breast Cancer

**DOI:** 10.1002/fsn3.71821

**Published:** 2026-04-30

**Authors:** Fabrice Tonfack Djikeng, Carister Nchangnwi, Jean Paul Chedjou, Hilaire Macaire Womeni

**Affiliations:** ^1^ Department of Biochemistry and Molecular Biology, Faculty of Science University of Buea Buea Cameroon; ^2^ Research Unit of Biochemistry, Medicinal Plants, Food Sciences, and Nutrition, Department of Biochemistry, Faculty of Science University of Dschang Dschang Cameroon

**Keywords:** African walnut oil, breast cancer, DHA, hematology, oxidative stress

## Abstract

This work analyzed the effect of African walnut oil (AWO) on tumor size, hematological and oxidative stress markers in female rats with 7,12‐dimethylbenz[a]anthracene (DMBA)‐induced breast cancer in comparison to docosahexaenoic acid (DHA). Eighteen rats with developed tumors were randomized into three groups of six rats each: the negative control, the positive control and test group. Six rats in which cancer was not induced served as the normal group. The four groups, respectively received distilled water (250 mg/kg), DHA (125 mg/kg), African walnut oil (1000 mg/kg), and distilled water by gavaging every day for 28 days. Tumor size was measured. The rats were sacrificed on the 29th day. The blood was collected, and part of it was used to prepare the serum. Organs of interest were used to prepare organ homogenates. Hematological markers were measured on the blood. The serum and organ homogenates were analyzed for their protein and oxidative stress markers. Results showed significantly (*p* < 0.001) higher tumor size in the negative control group (8.21 cm^3^) compared to the groups taking DHA (4 cm^3^) and AWO (5.37 cm^3^). Data from the hematological study showed that DHA and AWO oil increased white blood cells by 30.22%–88.83%, DHA increased platelets by 33.33% and AWO increased mean corpuscular hemoglobin in the blood by 32.94%. Rats that received DMBA presented significantly lower (*p* < 0.001) red blood cells (2.08–3.82 × 10^12^/L) and hemoglobin (5.10–7.30 g/dL) levels compared to the normal group (7.9 × 10^12^/L and 13.06 g/dL, respectively). The protein content of organ homogenate was significantly (*p* < 0.01) lower in the negative control group (0.16–2.92 g/dL) compared to other groups (~1.44–4.88 g/dL). Analysis of oxidative stress markers generally showed lower (*p* < 0.05) glutathione peroxidase, nitric oxide and catalase activities; and higher superoxide dismutase activity and malondialdehyde levels in the negative control groups. AWO oil could be used in delaying tumor growth, preserving hematological markers and limiting oxidative damage in rats with breast cancer.

## Graphical abstract

African walnut oil and docosahexaenoic acid administration to female Wistar rats with DMBA‐induced breast cancer may delay tumor growth, increase white blood cells, platelets and mean corpuscular hemoglobin counts in the blood. DMBA significantly lowers red blood cells, hemoglobin levels, and protein concentration. Docosahexaenoic acid and African walnut oil protect organs from the damages caused by oxidative stress.

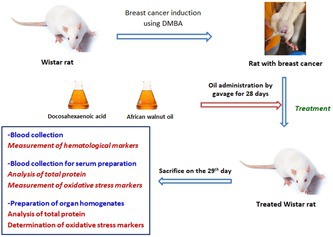

## 1 Introduction

Cancer is a disease that is characterized by uncontrolled cell division and proliferation, which can spread to other tissues (Akhouri et al. 2020). It is a leading cause of death worldwide, accounting for nearly 10 million deaths in 2020 with lung, prostate, colorectal, stomach and liver cancers being the most common types of cancer in men, whereas breast, colorectal, lung, cervical and thyroid cancers are the most common amongst women (World Health Organization 2022).

Breast cancer is the most often diagnosed cancer in women globally and the leading cause of death from cancer in them in over 100 nations (Bray et al. 2018). In 2022, there were 2.3 million women diagnosed with breast cancer and 670,000 deaths globally (World Health Organization 2022). According to Bray et al. (2024) report, there were close to 20 million new cancer cases and 9.7 million deaths globally, with lung and breast cancer being the most common. Sung et al. (2021) predicted an increase in the number of cancer cases to 35 million by 2050. Without action, the global cancer rate is expected to climb by 49% and fatalities by 62% by 2040 (Cao et al. 2021).

In Africa, breast cancer is the main cause of mortality amongst women, accounting for 28% of all cancers and 20% of all cancer‐related deaths in women (Ferlay et al. 2019). About 198,553 new cases of breast cancer were recorded with about 91,252 deaths in 2022 (Bray et al. 2024). In Cameroon, it remains a serious public health problem for women, with about 4170 (20.1%) new cases and 2108 (16%) deaths annually (Sung et al. 2021).

Breast cancer has been reported to be caused by several factors including age, genetic factors, reproductive issues, prolonged exposure to estrogen, not breastfeeding, and other lifestyle‐related factors, etc. In the same line, certain environmental factors also influence the development of breast cancer which includes tobacco smoking, grilled or smoked meat, and other lethal air pollutants including polycyclic aromatic hydrocarbons (PAHs), etc. (Obeagu and Obeagu 2024). Amongst the PAHs, 7,12‐dimethylbenz(a)anthracene (DMBA) is the most used to induce cancer in rats (Tatar et al. 2019). Exposure to this PAH was reported to increase the genesis of reactive oxygen species, overwhelming the antioxidant defense of the body and leading to damage of biomacromolecules (lipids, proteins, and DNA), cell dysfunction, abnormal cell development, and inflammation (Lai and Singh 2006).

Cancer has been associated with some homeostatic and hematological changes. It is known to induce the immune response that can modify hematological parameters such as white blood cells, red blood cells, hemoglobin, neutrophils, lymphocytes, monocytes, platelets, etc. as the cancer is progressing (Abbas et al. 2024; Berta et al. 2024). Oxidative stress is also known as playing an important role in cancer through the initiation and promotion of tumor growth by causing damages on the DNA molecules and mutations. Oxidative stress is also exploited by cancer cells to fuel their growth and survival when the concentration of reactive oxygen species is high (Iqbal et al. 2024). Breast cancer for example has been reported to be linked to altered antioxidant enzyme potential and cellular redox status. It is also associated to dysregulation of cell differentiation, excessive cell proliferation, incomplete apoptosis (Sahin et al. 2011). High concentration of reactive oxygen species, DNA alteration, reduction in antioxidant protection and lipid peroxidation have also be linked to breast cancer (Tas et al. 2005).

In order to manage this condition, several treatment options including chemotherapy, radiation, and surgery have been used. However, they are not accessible to all, are expensive and lead to various side effects (Vanderpuye et al. 2017). As a result, there is a growing interest in looking for natural ways to manage this condition. In this regard, natural plant extracts, omega‐3 fatty acids (docosahexaenoic acid especially) and other functional food ingredients from plant or animal origin have been tested and found efficient in managing and protecting against DMBA‐induced mammary carcinogenesis in rats (Mvondo et al. 2021; Sahin et al. 2011; Manna et al. 2011; Akhouri et al. 2020). Mvondo et al. (2021) reported that the aqueous extract of *Dacryodes edulis* leaves inhibits tumor growth in female rats with DMBA‐induced breast cancer. Akhouri et al. (2020) showed the therapeutic properties of *Aegle marmelos* fruit extract in DMBA‐induced breast cancer in rats. Sahin et al. (2011) demonstrated that a combination of lycopene and genistein offers maximum protection against DMBA‐induced mammary carcinogenesis in rats. In the same line, several studies have shown the efficiency of specific omega‐3 fatty acid against cancer. Omega‐3 fatty acids are polyunsaturated fatty acids which are essential for mammals and which were reported to have positive effects on several cancer types (Khankari et al. 2015). Amongst them, docosahexaenoic acid, a polyunsaturated fatty acid from fish oil and other marine sources, is the most popular in cancer science and has been demonstrated to have potent in vivo and in vitro anti‐tumor properties through various mechanisms in breast, leukemia, liver, prostate, lung, endometrial and gastric cancers (Earl et al. 2019). Early epidemiological evidence strongly linked fish oil (rich in docosahexaenoic acid and eicosapentaenoic acid) with low incidence in several types of cancer including breast cancer (Emad et al. 2022). Alqalshy et al. (2022) showed that docosahexaenoic acid has a chemo‐preventive effect in hamsters with DMBA‐induced buccal pouch cancer. Noguchi et al. (1997) demonstrated the chemo‐preventive potential of eicosapentaenoic acid and docosahexaenoic acid at low doses in rats with DMBA‐induced mammary cancer. Though several works have been done on the anticancer properties of docosahexaenoic acid, relatively few studies have explored the effect of omega‐3 fatty acids from plant origin (α‐linolenic acid) or oils obtained from plants rich in α‐linolenic acid against cancer induced in rat models using DMBA in therapeutic or preventive conditions. In one study, Zingue et al. (2024) reported that *Ricinodendron heudelotii* seed oil was efficient in preventing breast cancer in menopause‐like conditions DMBA‐induced cancer. It is therefore important to explore more plant sources of omega‐3 fatty acids that can be used as dietary supplements in the management and prevention of breast cancer and the physiological changes associated with it compared to docosahexaenoic acid, a well‐known nutraceutical with anti‐cancer activity.

African walnut ( *Tetracarpidium conophorum* ) is a non‐conventional oilseed highly available in Cameroon between August and September of each year and which is mostly consumed as a snack when boiled or roasted (Djikeng et al. 2017). It contains 55%–70% of edible oil. African walnut oil extracted from its seeds contains 5.70% saturated and 84.80% polyunsaturated fatty acids, with linoleic acid (14.41%) and α‐linoleic acid (70.39%) being the most represented (Ghomdim et al. 2024). The consumption of this oil has been associated with many health benefits including obesity, cardiovascular diseases, and type 2 diabetes management (Munteanu et al. 2025; Ghomdim et al. 2024; Douky et al. 2025). The efficiency of diets containing African walnut oil against prostate cancer in male rats was demonstrated by Uhunmwangho et al. (2022). On the other hand, Uhunmwangho et al. (2022) demonstrated that administration of diets containing *T. conophorum* oil significantly delayed the progression of 3‐Methylcholanthrene‐Induced Mammary Carcinogenesis in female Wistar rats. The anticancer activity of this oil is mostly attributed to its high content in α‐linolenic acid, an omega‐3 fatty acid (Yan et al. 2024). It will be important to investigate the effect of the administration of African walnut oil (from the nuts produced in Cameroon) by gavage on female Wistar rats with DMBA‐induced breast cancer and especially on tumor growth, hematological, and oxidative stress markers which are known to be influenced in cancer. This study was conducted in order to evaluate the effect of *T. conophorum* oil on tumor growth, hematological, and oxidative stress markers of female Wistar rats with 7,12‐dimethylbenz[a]anthracene (DMBA)‐Induced Breast Cancer in comparison to docosahexaenoic acid.

## 2 Materials and Methods

### 2.1 Materials

7,12‐Dimethylbenz[a]anthracene and docosahexaenoic acid (powder form) were purchased from Sigma‐Aldrich (Starnberg, Germany). All the other solvents and chemicals used were of analytical grade.

**FIGURE 1:**
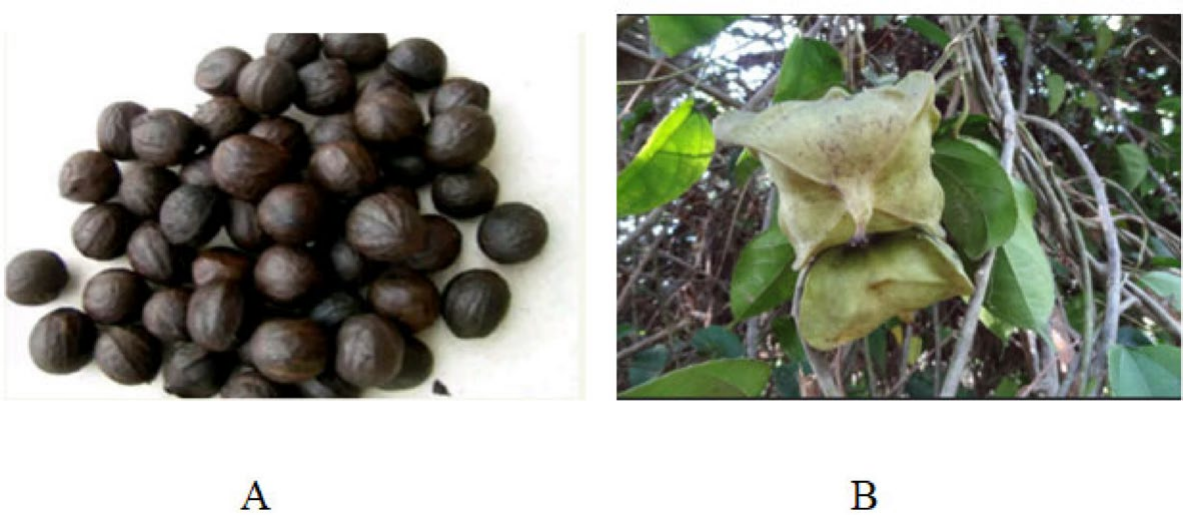
Shelled African walnut (A) and African walnut fruits on tree.

African walnuts (Figure 1) were purchased from a farmer in Essekou, Melong Littoral region of Cameroon in September 2024.

Fifty‐six female albino Wistar rats aged 6–9 weeks old weighing 55–60 g were purchased from a farmer in Yaoundé, Center Region of Cameroon.

### 2.2 Methods

#### 2.2.1 Extraction of African Walnut Oil

The extraction of the African walnut oil was done using the mixture methanol–chloroform–water (2:2:1) (Bligh and Dyer 1959). The nuts were washed with clean water to remove debris, sand, and other unwanted particles. Then the washed nuts were later dehulled (removal of the shell) and chopped into small sizes before the oil was extracted. About 100 g of African walnut was weighed and introduced in a blender and 200 mL of methanol was added. The mixture blended for 2 min, and 100 mL of chloroform was added. After blending for 2 min, another 100 mL of chloroform and 100 mL of distilled water were added. After proper mixing for 2 min, the solution was filtered using a muslin cloth, and the cake discarded. After that, the filtrate was again filtered using the whatman paper no. 1. The obtained filtrate was introduced into a funnel. The phase containing the mixture oil and chloroform was collected and evaporated using a rotary evaporator at 50°C under vacuum to eliminate the solvent. The extracted oil was transferred to a dark glass container and kept in the freezer at −18°C for further use.

#### 2.2.2 Animal Assays

##### 2.2.2.1 Ethical Clearance

All animals were cared for and used in agreement with international standard guideline for animal use. An ethical clearance for animal handling and care was obtained from the University of Buea Institutional Animal Care and Use Committee with the reference number UB‐IACUC No. 02/2025.

##### 2.2.2.2 Preparation of Animal Feed

A standard diet composed of corn‐meal (36.7%), wheat meal (36.6%), bone meal (14.5%), crushed fish (4.8%), palm kernel cake (7.3%), iodide salt (0.3%), and vitamin (0.01%) (Zingue et al. 2024).

##### 2.2.2.3 Animal Grouping and Feeding

For this study, 56 female albino Wistar rats aged 6–9 weeks (55–60 g) were used. The rats were allowed to acclimatize under a 12‐h light–dark cycle at room temperature for 7 days in cages containing sawdust, with ad libitum access to food and water prior to the start of the experiment. Out of the 56 rats, 50 were anesthetized (using 10 mg/kg diazepam and 50 mg/kg ketamine intraperitoneally) and cancer induced in them by injecting each of them with a single dose (50 mg/kg BW) of DMBA dissolved in 1 mL olive oil via subcutaneous intra‐mammary route (Zingue et al. 2024). The six other rats that were not induced served as the normal group. To avoid infection, all DMBA‐induced rats received 89 mg/kg of amoxicillin by gavage for 10 days while the normal group received distilled water. The rats were palpated after every 2 days to check if the tumor was developing. Breast tumor started appearing and being palpable around the 19th week. Rats with palpable tumors (18) were randomized into three groups of six rats each plus the normal group (06) for a total of 24 rats. Betadine and penicillin ointment were used to clean and treat the rats in case of any wounds twice daily. The positive control group was given docosahexaenoic acid (125 mg/kg) (El‐Mesery et al. 2009) while the test group was given *T. conophorum* oil (1000 mg/kg) by gavaging every day for 28 days (Table 1) (Djikeng et al. 2025). The negative control and the normal groups were given water. The tumor size was measured using a caliper after every 2 days to check for tumor reduction and its volume (length, width, and height) calculated using the formula below. On the 28th day, the rats were allowed to fast overnight and they were sacrificed on the 29th day.

$$\frac{\pi }{6}\times \text{length}\times \text{width}\times \text{height}$$

**TABLE 1:** Animal group distribution and characteristics.

Groups	Characteristics
Group 1 (normal)	Normal rats receiving distilled water (250 mg/kg BW)
Group 2 (negative control)	Rats with cancer receiving distilled water (250 mg/kg BW)
Group 3 (positive control)	Rats with cancer receiving docosahexaenoic acid at a dose of 125 mg/kg BW
Group 4 (test group)	Rats with cancer receiving oral administration of 1000 mg/kg BW of *Tetracarpidium conophorum* oil daily for 28 days by gavaging

##### 2.2.2.4 Animal Sacrifice and Sample Collection

The animals were sacrificed by incision of the abdominal artery on the 29th day after they were allowed to fast overnight. Before the sacrifice, they were anesthetized through intraperitoneal injection of Ketamine 60 mg/kg and Diazepam 10 mg/kg of body weight. Blood was collected by cardiac puncture using a 5 mL syringe. Part of the blood was introduced in tubes with EDTA and used for the analysis of hematological parameters while the other part was introduced in tubes without EDTA and used for the preparation of the serum. The serum was obtained by centrifuging the blood at 3500 rpm for 15 min. The organs of interest (heart, kidneys, pancreas, brain, tumor from the mammary gland, and liver) were collected, weighed, and used for the preparation of organ homogenates. Tumor size was also measured. Organ homogenates were prepared in tris HCl (20 g of organ/100 mL of tris HCl). The organ was crushed in the solvent and the mixture centrifuged at 3500 rpm for 10 min. The obtained serum and organ homogenates were used for the determination of oxidative stress markers.

##### 2.2.2.5 Measurement of Hematological Markers

An automatic hematological analyzer (SFRI H18 LIGHT auto Hematology Analyzer) was used to analyze the blood samples. The following markers were measured: White blood cells, lymphocytes, granulocytes, red blood cells, hemoglobin, hematocrit, mean corpuscular volume, mean corpuscular hemoglobin, and platelets were measured (Djikeng et al. 2025).

##### 2.2.2.6 Determination of the Serum and Organ Homogenates Protein Content

The liquid reagent method described by the SGM kit was used for the determination of the total protein. In a test tube, 1000 μL of reagent 1 was added, followed by the addition of 10 μL of distilled water or sample or standard. After that, the mixture was stirred and allowed to incubate for 10 min at 37°C. The absorbances of sample (*A*_x_) and standard (*A*_s_) were recorded against the blank at 540 nm. The total protein was calculated as follows:

$$\text{Total protein}\;\left(g/\mathrm{dL}\right)=A_{x}/A_{s}\times \mathrm{STD}\;\text{concentration}$$

##### 2.2.2.7 Determination of Oxidative Stress Markers

The organ homogenates and serum collected were analyzed for oxidative stress parameters. The superoxide dismutase (SOD) was measured according to the method described by Oberley et al. (1998). The malondialdehyde concentration was determined following the method of Wilbur et al. (1990). The nitric oxide level was assayed as described by Montgomery et al. (1961). The catalase activity was evaluated using the protocol of Sinha (1972). The reduced glutathione peroxidase was analyzed using the method described by Ellman (1959).

##### 2.2.2.8 Statistical Analysis

*n* = 6. Data were subjected to one‐way analysis of variance (ANOVA). The Student–Newman–Keuls test was used to appraise the statistical significance of the data using the software Statgraphics Centurion version XVI in order to evaluate the statistical significance of the data. A probability value at *p* < 0.05 was considered to be statistically significant.

## 3 Results

### 3.1 Animal Assays

#### 3.1.1 Organ Weight

The organ weight of animals after they were sacrificed is presented in Table 2. No significant (*p* > 0.05) difference was recorded in the organ weights of the liver, pancreas, right kidney, left kidney, and brain. Rats taking African walnut oil and DHA exhibited the lowest (*p* < 0.05) heart weights compared to the negative control group and the normal group.

**TABLE 2:** Weight of organs (g).

Rat groups	Liver	Pancreas	Left kidney	Right kidney	Brain	Heart
Normal group	7.92 ± 0.53^a^	1.60 ± 0.43^a^	0.69 ± 0.01^a^	0.76 ± 0.00^a^	1.70 ± 0.02^a^	0.90 ± 0.03^a^
Negative control (DMBA alone)	7.50 ± 1.27^a^	0.45 ± 0.22^a^	0.77 ± 0.13^a^	0.92 ± 0.24^a^	1.46 ± 0.28^a^	0.91 ± 0.11^a^
DMBA + docosahexaenoic acid	7.23 ± 0.47^a^	0.70 ± 0.15^a^	0.70 ± 0.09^a^	0.71 ± 0.14^a^	1.51 ± 0.11^a^	0.69 ± 0.05^b^
DMBA + African walnut oil	6.21 ± 0.17^a^	0.87 ± 0.11^a^	0.57 ± 0.07^a^	0.57 ± 0.04^a^	1.13 ± 0.12^a^	0.54 ± 0.09^c^

*Note:* *n* = 6. Values are presented as mean ± standard deviation. Values of the same column with different superscripts (a–c) are significantly different at *p* < 0.05. Normal: Rats without cancer that received distilled water; DMBA + docosahexaenoic acid: Rats with breast cancer treated with docosahexaenoic; Negative control: Rats with breast cancer and untreated; DMBA + African walnut oil: Rats with breast cancer treated with African walnut oil.

#### 3.1.2 Average Tumor Size and Morphology

**FIGURE 2:**
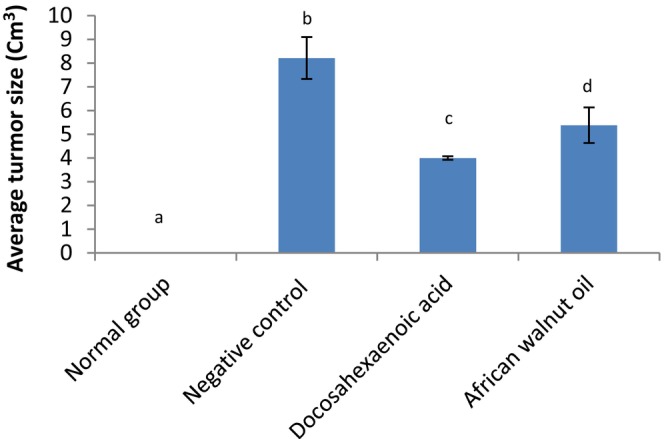
Average tumor size (Normal: Rats without cancer that received distilled water; DMBA + docosahexaenoic acid: Rats with breast cancer treated with docosahexaenoic; Negative control: Rats with breast cancer and untreated; DMBA + African walnut oil: Rats with breast cancer treated with African walnut oil). *n* = 6. Values are presented as mean ± standard deviation. Values with different superscripts (a–d) are significantly different at *p* < 0.01.

The average tumor size of animals throughout the experiment showed that the negative control group presented a significantly (*p* < 0.001) higher tumor size compared to the other groups. It was followed by the group taking *T. conophorum* oil. The group taking docosahexaenoic acid had the lowest tumor size. No tumor was recorded in the normal group (Figure 2).

**FIGURE 3:**
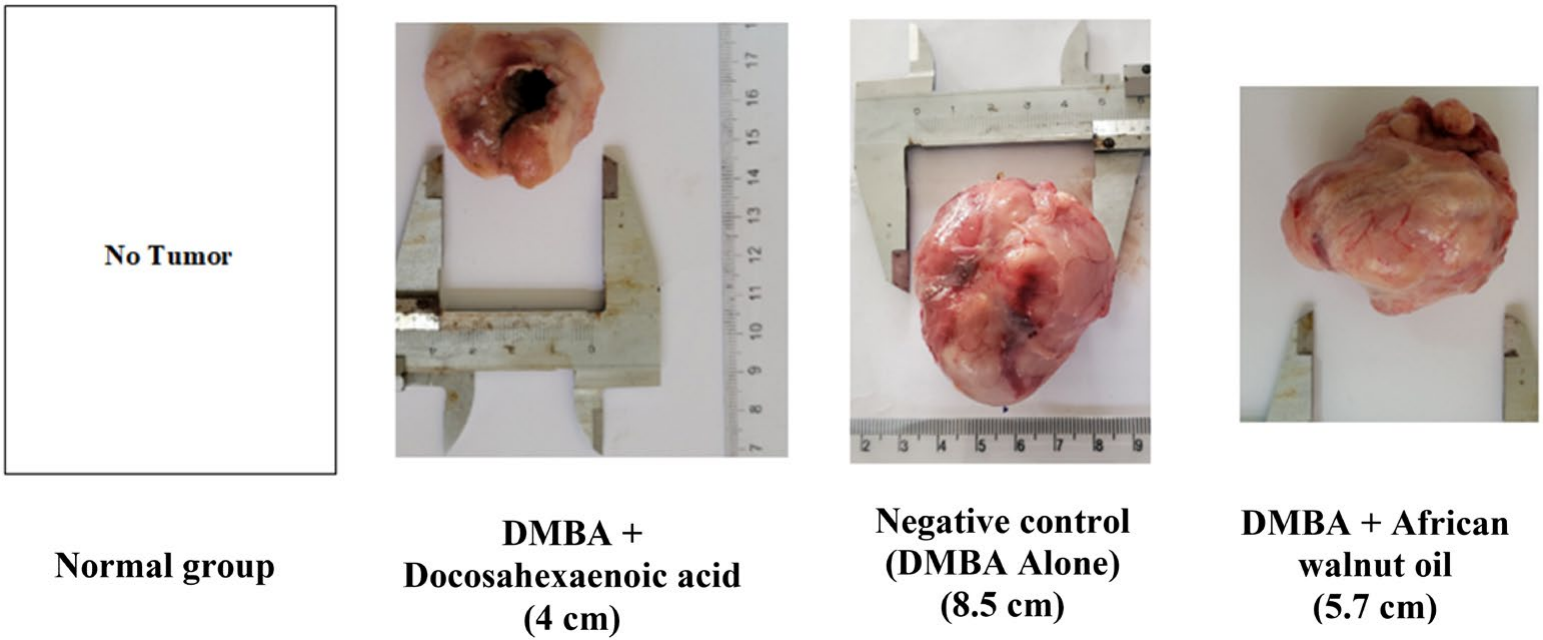
Tumor morphology (Normal: Rats without cancer that received distilled water; DMBA + docosahexaenoic acid: Rats with breast cancer treated with docosahexaenoic acid; Negative control: Rats with breast cancer and untreated; DMBA + African walnut oil: Rats with breast cancer treated with African walnut oil).

The highest tumor was developed by the negative control group (8.5 cm). It was followed by the group taking African walnut oil (5.7 cm) and the one taking DHA (4 cm). Meanwhile, no tumor was recorded in the normal group (Figure 3).

#### 3.1.3 Effect of African Walnut Oil and DHA on Hematological Parameters

The effect of African walnut oil and docosahexaenoic acid on hematological parameters of DMBA‐induced breast cancer in female Wistar rats is presented in Table 3. The rats that received the DMBA exhibited a significantly (*p* < 0.05) higher WBC% compared to the normal group. The highest (*p* < 0.05) WBC was noted in the groups that received African walnut oil and DHA. The negative control and the group taking African walnut oil presented the lowest lymphocytes% compared to the rats of the normal group and those taking DHA. However, it is important to note that the negative control group presented the least (*p* < 0.05) lymphocytes%. Looking at the MID%, the negative control group presented the highest (*p* < 0.05) value compared to the normal group and the group taking *T. conophorum* oil and docosahexaenoic acid. For the GRAN%, the negative control group and the group taking African walnut oil presented significantly (*p* < 0.001) higher values compared to the other groups. The rats of the normal group presented the highest RBC, HGB and HCT% compared to those that received the DMBA. Concerning the MCV%, the negative control group presented the lowest (*p* < 0.05) values compared to the other groups in which this parameter remained non‐significant (*p* > 0.05). The rats taking African walnut oil showed significantly (*p* < 0.01) higher MCH and MCHC% compared to those of the other groups. Looking at the platelets, the lowest (*p* < 0.01) value was recorded with the negative control group compared to the normal group and those taking DHA and African walnut oil.

**TABLE 3:** Effect of African walnut oil and docosahexaenoic acid on hematological parameters in DMBA‐induced breast cancer Wistar rats.

	WBC (10^9^/L)	LYMP (%)	GRAN (%)	RBC (10^12^/L)	HGB (g/dL)
Normal group	9.40 ± 1.11^a^	76.96 ± 2.11^a^	7.56 ± 0.90^a^	7.39 ± 0.30^a^	13.06 ± 0.87^a^
Negative control (DMBA Alone)	12.25 ± 0.35^b^	45.83 ± 1.55^b^	33.40 ± 1.27^b^	3.58 ± 0.16^b^	5.56 ± 0.23^b^
DMBA + docosahexaenoic acid	17.75 ± 1.01^c^	74.40 ± 4.30^a^	8.20 ± 0.12^a^	3.82 ± 0.50^b^	7.30 ± 1.55^b^
DMBA + African walnut oil	14.50 ± 0.02^d^	52.00 ± 1.33^c^	33.60 ± 3.33^b^	2.08 ± 0.45^b^	5.10 ± 0.75^b^

	HCT (%)	MCV (fL)	MCH (pg)	MCHC (g/dL)	PLT (10^9^/L)
Normal group	36.93 ± 4.25^a^	56.88 ± 0.95^ab^	18.43 ± 0.76^a^	32.25 ± 0.44^a^	423.00 ± 20.11^a^
Negative control (DMBA alone)	19.85 ± 0.07^b^	53.74 ± 1.05^b^	15.50 ± 0.10^a^	29.00 ± 0.60^a^	138.00 ± 4.24^b^
DMBA + docosahexaenoic acid	23.25 ± 1.67^c^	56.25 ± 3.46^ab^	17.55 ± 0.49^a^	33.10 ± 1.55^a^	564.00 ± 63.63^c^
DMBA + African walnut oil	12.20 ± 2.41^d^	59.10 ± 0.13^a^	24.50 ± 0.01^b^	41.80 ± 1.22^a^	179.00 ± 22.55^d^

*Note:* *n* = 6. Values are presented as mean ± standard deviation. Values of the same column with different superscripts (a–d) are significantly different at *p* < 0.05. Normal: Rats without cancer that received distilled water; DMBA + docosahexaenoic acid: Rats with breast cancer treated with docosahexaenoic; Negative control: Rats with breast cancer and untreated; DMBA + African walnut oil: Rats with breast cancer treated with African walnut oil.

#### 3.1.4 Effect of African Walnut Oil and DHA on Total Protein

The variation in total protein of tumor, organ homogenates and serum is presented in Table 4. The rats in the negative control group exhibited significantly (*p* < 0.05) lower protein levels in the tumor, brain, pancreas, right kidneys, liver and heart compared to the other groups. Meanwhile, at the level of the left kidney, the negative control and the group receiving African walnut oil exhibited significantly (*p* < 0.05) lower protein levels compared to the group taking DHA and the normal group. No significant (*p* > 0.05) difference in total protein was recorded in the serum of all groups.

**TABLE 4:** Total protein (g/dL) of serum, tumor and organs homogenates of rats.

	Tumor	Brain	Pancreas	Right kidney	Left kidney	Liver	Heart	Serum
Normal group	ND	2.07 ± 1.25^a^	2.05 ± 0.07^a^	3.46 ± 0.60^a^	3.46 ± 0.23^a^	4.03 ± 0.00^a^	2.86 ± 0.39^a^	8.65 ± 1.07^a^
Negative control (DMBA alone)	0.40 ± 0.00^b^	0.22 ± 0.00^b^	0.16 ± 0.02^b^	0.92 ± 0.00^b^	2.92 ± 0.00^b^	0.42 ± 0.13^b^	0.44 ± 0.00^b^	5.97 ± 0.69^a^
DMBA + docosahexaenoic acid	3.81 ± 0.00^c^	4.77 ± 0.00^c^	2.90 ± 0.60^a^	3.27 ± 0.75^a^	5.32 ± 0.00^c^	3.03 ± 0.00^c^	4.88 ± 0.00^c^	6.11 ± 1.09^a^
DMBA + African walnut oil	2.44 ± 0.00^d^	1.96 ± 0.00^a^	2.55 ± 0.00^a^	2.33 ± 0.00^c^	2.40 ± 0.00^b^	1.44 ± 0.00^d^	2.55 ± 0.00^a^	6.43 ± 0.00^a^

*Note:* *n* = 6. Values are presented as mean ± standard deviation. Values of the same column with different superscripts (a–d) are significantly different at *p* < 0.05. Normal: Rats without cancer that received distilled water; DMBA + docosahexaenoic acid: Rats with breast cancer treated with docosahexaenoic; Negative control: Rats with breast cancer and untreated; DMBA + African walnut oil: Rats with breast cancer treated with African walnut oil.

#### 3.1.5 Effect of African Walnut Oil and DHA on Oxidative Stress Markers

##### 3.1.5.1 Superoxide Dismutase

The SOD activities of organ, tumor homogenates and serum of rats are shown in Table 5. The negative control group showed significantly (*p* < 0.05) higher SOD activity across all organs and serum when compared to the other groups.

**TABLE 5:** Variations in SOD activity (U/mg of protein) of serum, tumor and organ homogenates of rats.

	Tumor	Brain	Pancreas	Right kidney	Left kidney	Liver	Heart	Serum
Normal group	ND	0.54 ± 0.05^a^	0.29 ± 0.00^a^	0.23 ± 0.03^a^	0.17 ± 0.00^a^	0.35 ± 0.00^a^	0.10 ± 0.00^a^	0.16 ± 0.02^a^
Negative control (DMBA alone)	24.71 ± 0.67^b^	10.40 ± 3.06^b^	10.65 ± 0.96^b^	2.02 ± 0.07^b^	23.83 ± 1.13^b^	4.15 ± 0.92^b^	1.15 ± 0.01^b^	0.32 ± 0.02^b^
DMBA + docosahexaenoic acid	0.47 ± 0.00^c^	0.30 ± 0.02^c^	0.45 ± 0.00^c^	0.47 ± 0.00^c^	0.31 ± 0.00^c^	0.44 ± 0.00^c^	0.24 ± 0.00^c^	0.28 ± 0.03^bc^
DMBA + African walnut oil	0.68 ± 0.00^d^	0.71 ± 0.00^d^	0.60 ± 0.00^d^	0.76 ± 0.00^d^	0.58 ± 0.00^d^	1.13 ± 0.00^d^	0.62 ± 0.00^d^	0.26 ± 0.00^c^

*Note:* *n* = 6. Values are presented as mean ± standard deviation. Values of the same column with different superscripts (a–d) are significantly different at *p* < 0.05. Normal: Rats without cancer that received distilled water; DMBA + docosahexaenoic acid: Rats with breast cancer treated with docosahexaenoic; Negative control: Rats with breast cancer and untreated; DMBA + African walnut oil: Rats with breast cancer treated with African walnut oil.

##### 3.1.5.2 Reduced Glutathione Peroxidase

The changes in glutathione activity of tumor, organ homogenates and serum of rats are presented in Table 6. The negative control group presented significantly (*p* < 0.05) lower GSH activities in the brain, pancreas, left kidney and liver, respectively compared to the other groups. Looking at the tumor, the highest (*p* < 0.001) GSH activity was observed in the rat group taking African walnut oil. At the level of the right kidney, the group taking African walnut oil presented significantly (*p* < 0.001) lower GSH activity compared to other groups. At the level of the heart, the normal and the negative control groups presented significantly (*p* < 0.05) higher GSH activities compared to the groups. There was no significant (*p* > 0.05) difference in GSH activity of the serum across all groups.

**TABLE 6:** Glutathione (GSH) peroxidase level (μmol) of serum, tumor and organs homogenates of rats.

	Tumor	Brain	Pancreas	Right kidney	Left kidney	Liver	Heart	Serum
Normal group	ND	11.69 ± 0.00^a^	12.96 ± 0.00^a^	21.09 ± 0.50^a^	22.50 ± 1.17^a^	17.97 ± 1.27^a^	21.45 ± 2.14^a^	22.65 ± 1.55^a^
Negative control (DMBA alone)	5.01 ± 1.95^b^	5.85 ± 0.70^b^	6.80 ± 3.14^b^	14.11 ± 1.83^b^	14.93 ± 3.55^b^	8.00 ± 0.02^b^	21.55 ± 0.27^a^	23.52 ± 1.22^a^
DMBA + docosahexaenoic acid	3.15 ± 0.00^b^	8.02 ± 0.75^c^	24.77 ± 0.00^c^	18.03 ± 0.50^c^	20.03 ± 0.00^ab^	21.50 ± 0.00^c^	11.58 ± 0.00^b^	22.22 ± 0.73^a^
DMBA + African walnut oil	14.30 ± 0.00^c^	15.62 ± 0.00^d^	14.18 ± 0.00^a^	8.03 ± 0.00^d^	15.72 ± 0.00^b^	16.483 ± 0.00^a^	18.90 ± 0.00^c^	20.84 ± 2.94^a^

*Note:* *n* = 6. Values are presented as mean ± standard deviation. Values of the same column with different superscripts (a–d) are significantly different at *p* < 0.05. Normal: Rats without cancer that received distilled water; DMBA + docosahexaenoic acid: Rats with breast cancer treated with docosahexaenoic; Negative control: Rats with breast cancer and untreated; DMBA + African walnut oil: Rats with breast cancer treated with African walnut oil.

##### 3.1.5.3 Nitric Oxide

The NO levels in tumor, organ homogenates and serum of rats are presented in Table 7. The negative control group showed significantly (*p* < 0.05) lower NO levels in the tumor and brain when compared to the other groups. At the level of the left kidney, right kidney and heart, the group receiving DHA and the normal group both presented significantly (*p* < 0.05) higher NO levels. The group receiving African walnut oil showed the lowest NO level in these organs. In the liver, the group receiving African walnut oil and negative control both exhibited the lowest (*p* < 0.05) NO levels compared to the normal and DHA groups. The negative control group showed significantly (*p* < 0.05) higher NO level compared to the other groups. The lowest NO concentration in the serum was recorded with the group taking African walnut oil.

**TABLE 7:** Nitric oxide level (μM/L) of serum, tumor and organs homogenates of rats.

	Tumor	Brain	Pancreas	Right kidney	Left kidney	Liver	Heart	Serum
Normal group	ND	12.73 ± 0.00^a^	16.70 ± 0.00^a^	17.83 ± 1.30^a^	21.59 ± 0.00^a^	5.63 ± 0.34^a^	14.98 ± 0.00^a^	1.41 ± 0.21^ab^
Negative control (DMBA alone)	1.81 ± 0.08^b^	3.46 ± 0.64^b^	19.50 ± 3.96^a^	12.00 ± 0.00^a^	13.69 ± 1.18^b^	2.22 ± 0.17^b^	9.89 ± 1.39^b^	2.32 ± 0.49^bc^
DMBA + docosahexaenoic acid	5.36 ± 0.00^c^	14.40 ± 0.00^a^	26.65 ± 3.97^b^	31.21 ± 0.00^b^	27.34 ± 0.00^c^	8.98 ± 0.00^c^	14.70 ± 0.00^a^	4.65 ± 0.32^c^
DMBA + African walnut oil	11.41 ± 0.00^d^	6.25 ± 0.00^c^	5.97 ± 0.00^c^	1.97 ± 0.00^c^	5.41 ± 0.00^d^	2.81 ± 0.00^b^	4.83 ± 0.00^c^	0.78 ± 0.00^a^

*Note:* *n* = 6. Values are presented as mean ± standard deviation. Values of the same column with different superscripts (a–d) are significantly different at *p* < 0.05. Normal: Rats without cancer that received distilled water; DMBA + docosahexaenoic acid: Rats with breast cancer treated with docosahexaenoic; Negative control: Rats with breast cancer and untreated; DMBA + African walnut oil: Rats with breast cancer treated with African walnut oil.

##### 3.1.5.4 Catalase Activity

The catalase activities of the tumor, organ homogenates and serum are shown in Table 8. The group that received *T. conophorum* exhibited the highest CAT level in the tumor when compared to the normal and the group receiving DHA. The group receiving DHA showed a significantly (*p* < 0.05) higher catalase activity in the brain, right kidney, and liver when compared to the other groups. Meanwhile, at the level of the pancreas, left kidney and heart, the normal group had the highest (*p* < 0.05) catalase activity and this was closely followed by the group receiving DHA. For the serum, the negative control group exhibited a significantly (*p* < 0.05) higher catalase activity compared to the other groups.

**TABLE 8:** Catalase activity (μmol H_2_O_2_/min/mg) of serum, tumor and organs homogenates of rats.

	Tumor	Brain	Pancreas	Right kidney	Left kidney	Liver	Heart	Serum
Normal group	ND	22.08 ± 2.29^a^	21.40 ± 0.60^a^	29.78 ± 0.84^a^	31.15 ± 0.84^a^	25.02 ± 2.66^a^	25.45 ± 4.55^a^	45.32 ± 5.88^a^
Negative control (DMBA alone)	14.62 ± 0.04^b^	14.30 ± 1.28^b^	17.15 ± 3.18^b^	11.42 ± 2.33^b^	11.97 ± 0.33^b^	14.04 ± 2.14^b^	10.34 ± 1.41^b^	89.20 ± 7.25^c^
DMBA + docosahexaenoic acid	13.45 ± 0.00^b^	41.15 ± 0.00^c^	20.12 ± 0.00^a^	46.45 ± 0.00^c^	29.35 ± 0.00^a^	27.01 ± 0.00^a^	20.23 ± 0.00^c^	52.35 ± 2.86^b^
DMBA + African walnut oil	18.63 ± 0.00^c^	22.51 ± 0.00^a^	15.84 ± 0.00^b^	10.88 ± 0.00^b^	11.62 ± 0.00^b^	14.59 ± 0.00^a^	12.25 ± 0.00^b^	20.97 ± 0.00^a^

*Note:* *n* = 6. Values are presented as mean ± standard deviation. Values of the same column with different superscripts (a–c) are significantly different at *p* < 0.05. Normal: Rats without cancer that received distilled water; DMBA + docosahexaenoic acid: Rats with breast cancer treated with docosahexaenoic; Negative control: Rats with breast cancer and untreated; DMBA + African walnut oil: Rats with breast cancer treated with African walnut oil.

##### 3.1.5.5 Malondialdehyde Level

The MDA levels of serum, tumor and organ homogenates of the rats are presented in Table 9. The negative control group showed the highest (*p* < 0.05) MDA levels in the tumor, organs and serum across all groups.

**TABLE 9:** Malondialdehyde levels (μmol/L) of serum, tumor, and organs homogenates of rats.

	Tumor	Brain	Pancreas	Right kidney	Left kidney	Liver	Heart	Serum
Normal group	ND	8.23 ± 2.05^a^	4.27 ± 1.20^a^	10.74 ± 0.09^a^	10.19 ± 2.72^a^	8.06 ± 0.30^a^	9.96 ± 0.00^a^	2.86 ± 0.14^b^
Negative control (DMBA alone)	9.80 ± 0.42^b^	14.48 ± 1.14^b^	14.22 ± 0.78^b^	17.48 ± 1.48^b^	17.11 ± 1.28^b^	16.13 ± 0.79^b^	16.28 ± 1.49^b^	11.71 ± 0.00^c^
DMBA + docosahexaenoic acid	1.55 ± 0.00^c^	8.87 ± 3.84^a^	4.30 ± 1.11^a^	4.18 ± 0.00^c^	5.23 ± 0.00^c^	5.94 ± 0.00^c^	2.97 ± 1.18^c^	1.93 ± 0.41^ab^
DMBA + African walnut oil	6.49 ± 0.00^d^	7.77 ± 0.00^a^	6.33 ± 0.00^c^	7.77 ± 0.00^d^	9.07 ± 0.00^d^	6.46 ± 0.00^d^	6.34 ± 0.00^d^	1.28 ± 0.00^a^

*Note:* *n* = 6. Values are presented as mean ± standard deviation. Values of the same column with different superscripts (a–d) are significantly different at *p* < 0.05. Normal: Rats without cancer that received distilled water; DMBA + docosahexaenoic acid: Rats with breast cancer treated with docosahexaenoic; Negative control: Rats with breast cancer and untreated; DMBA + African walnut oil: Rats with breast cancer treated with African walnut oil.

## 4 Discussion

### 4.1 Tumor Size and Morphology

Tumor size is a key determinant of cancer progression and therapy success. In this study, the negative control group (DMBA‐induced but untreated) had the greatest tumor mass, demonstrating DMBA's powerful capacity to initiate and promote breast tumor formation and disruption in cell proliferation. This is in line with research carried out by Zingue et al. (2024) who made a similar observation with ovariectomized Wistar rats with DMBA‐induced cancer. The group taking African walnut oil showed a moderate reduction in tumor size. This shows a partial protective impact, most likely due to the oil's high amount of polyunsaturated fatty acids, antioxidant and antiproliferative effects Vilakazi et al. (2021). Zingue et al. (2024) also recorded a reduction in tumor size in DMBA‐induced breast cancer in the rats receiving the “Njangsang” oil when compared to the rats that were not taking any oil. The group taking DHA had the smallest tumor size compared to the other DMBA‐induced groups, confirming its effectiveness in inhibiting tumor development. It has been reported that docosahexaenoic acid has the potential to inhibit tumor development by boosting apoptosis, reducing cell proliferation, cell migration and invasion in several cancer types. It is suspected that these effects are facilitated through mechanisms such as induction of reactive oxygen species, inactivating the PI_3_K/Akt and MAPK signaling pathways and modulating microRNAs like miR‐138‐5p (Bai et al. 2019; West et al. 2020; Bilyk et al. 2022). According to Uhunmwangho et al. (2022), the main mechanisms used by African walnut oil against cancer comprise the modulation of COX‐2 and peroxisome proliferator‐activated receptor gamma signaling pathways. The oil was reported to significantly diminish COX‐2 expression, which is frequently upregulated in cancerous tissues and linked with inflammation and tumor development (Uhunmwangho et al. 2022). African walnut oil was also reported to increase peroxisome proliferator activated receptor gamma activity, suggesting an anti‐inflammatory effect that contributes to its anticancer properties. The oil has good polyunsaturated fatty acids content, especially linolenic acid, which is related to tumor growth inhibition and cancer cell death through toxicity (Chen et al. 2025).

### 4.2 Organs Weights

Organ weight assessment is a typical metric used in experimental animal investigations to evaluate possible toxicity, organ‐specific effects, and systemic influence of test chemicals. There were no significant variations in the weights of the liver, pancreas, kidneys (right and left), and brain between the experimental groups in this investigation. This consistency implies that neither DMBA treatment nor the therapies (African walnut oil and DHA) caused obvious toxicity or organ hypertrophy/atrophy in these organs. Additionally, the groups taking African walnut oil and DHA had lower heart weights than the negative control and normal groups. Although a decrease in heart weight may raise immediate worries, it could be due to a decrease in systemic inflammation, oxidative stress, or lipid accumulation—all of which are documented side effects of DMBA toxicity (Abd El‐Kader and Saiem Al‐Dahr 2016).

### 4.3 Hematological Markers

The hematological outcomes of this investigation demonstrated that DMBA‐induced groups had considerably higher WBC counts which are above the normal range (3.5–10.0 × 10^9^/L) for normal white blood cell count compared to the normal group. The group taking DHA exhibited the highest WBC level. This is contradictory to the previous reports that showed that docosahexaenoic acid suppresses tumor development and attenuates changes in the immune system in breast cancer models rather than increasing WBC in a stimulatory way (Munhoz et al. 2025). High WBC count can be an indication of infection, inflammation and immune suppression (Chmielewski and Strzelec 2018). In rats with tumors, elevated WBCs indicate an inflammatory response, which raises the likelihood of developing invasive breast cancer (Park et al. 2019). This is in line with the work done by Emam et al. (2022) who assessed the role of wheat germ oil in the prevention of DMBA‐induced breast cancer in rats and recorded a high WBC in the DMBA‐induced groups compared to the normal control group. The high WBC value of the groups taking African walnut and DHA might therefore be the consequence of DMBA administration. Reports showed that DMBA's carcinogenic effect and the appearance of inflammation can affect the immune response leading to an increase or decrease in WBC.

The negative control group had the lowest lymphocyte % compared to the rats of the normal group and those from the groups taking DHA and African walnut oil. Lymphocytes play a vital role in fighting cancer, since low lymphocyte numbers are related to relapse and poor survival rates, immune suppression, whereas greater counts enhance overall survival and immune activation (Akinbami et al. 2013). It has been reported that omega‐3 fatty acids such as DHA have a systemic effect on immune function by preventing T‐cell depletion and maintaining immune cell concentrations during chemotherapy treatments (Munhoz et al. 2025).

For the platelet, the negative control group had the lowest platelet count (138 × 10^9^/L) when compared to the normal group and the groups that received DHA and African walnut oil. A low platelet count is linked to bone marrow suppression, platelet destruction as a result of cancer progression (Rochet et al. 2015). This is related to the production of growth factors and cytokines that stimulate angiogenesis, a crucial stage in breast cancer spreading (Etim et al. 2018).

The rats of the normal group presented the highest RBC, HCB, and HCT% compared to those that received the DMBA. Normal red blood cell count is 12.1–15.1 g/dL and the DMBA‐induced groups had a RBC count of less than 5 g/dL. The low RBC, HGB, and HCT level can be attributed to the tumor development that generally leads in cancer patients to anemia. Cancer often causes anemia and inflammation/chronic disease due to iron deficiency, as cancer can reduce red blood cell synthesis or interact with iron storage cells, resulting in decreased iron absorption (Gaspar et al. 2015). This is in accordance with the work done by Emam et al. (2022). Previous reports showed that RBC, HGB, and HTC levels in rats with breast cancer significantly decrease compared to the normal group leading to anemia. In one study, Bregolat et al. (2022) showed that TF mice with breast cancer exhibited significant drops in HBC, RBC, and HTC compared to the normal group. This supports the data obtained in this study.

Looking at the MCHC, results revealed no significant difference in its concentration amongst the groups while the MCH level was high in the group taking African walnut oil compared to the other groups. Some research work on humans with breast cancer has revealed that MCH and MCHC levels tend to be lower especially in case of the presence of anemia but these vary across various works and rats (Chinedu‐Madu Jane et al. 2025). Some human studies link high MCH with worse results and higher HER2‐positive tumors. At the same time, in some studies, MCH has no statistical consideration or decreases in breast cancer groups. Data on rats and MCH in breast cancer studies is still limited (Zhang et al. 2016).

### 4.4 Serum Protein Level

In the current study, the negative control group had significantly lower total protein levels in the tumor, brain, pancreas, right kidney, liver, and heart as compared to the other groups. This finding is consistent with prior research showing that DMBA‐induced carcinogenesis causes increased protein catabolism and decreased protein synthesis, which is commonly caused by oxidative stress and tissue damage (Shivani et al. 2025). Meanwhile, the groups that received DHA and African walnut oil maintained considerably greater protein levels across all organs and serum examined, which is consistent with omega‐3 fatty acids' known roles in preserving cellular membranes, enhancing protein synthesis, and lowering inflammation (Calviello et al. 2007). DHA may achieve these effects by influencing transcription factors involved in cellular repair and antioxidant response, reducing tissue damage and protein loss (Díaz et al. 2021). This is similar to the work done by Pratama et al. (2024) on the proteomic effect of mammary carcinogenesis induced by DMBA on rats where the total protein concentration in the test group was increased by 27% compared to that in the negative control group.

### 4.5 Effect of Oil on Oxidative Stress Makers

Oxidative stress contributes significantly to disease development. MDA, SOD, NO, GSH, and CAT levels were measured in serum and organ homogenates to determine how African walnut oil and docosahexaenoic acid, both high in omega 3, helped manage breast cancer in Wistar rats exposed to the environmental carcinogen DMBA. SOD eliminates ROS enzymatically (Park et al. 2019). Catalase reduces hydrogen peroxides and protects tissues from reactive hydroxyl radicals (Rasheed 2024). DMBA causes tumorigenesis by producing ROS, which promotes cancer (Wang and Zhang 2017). The present study showed that DMBA increased oxidative stress in cancer‐induced rats through the elevated MDA levels exhibited by the negative control group at the level of the tumor, organs and serum when compared to the normal group. According to Rasic et al. (2018), serum MDA levels are extremely higher in cancer patients than in normal patients. This result is in line with those of Rasheed (2024) who found that DMBA increases MDA levels and decreases antioxidant enzymes due to increased ROS production. This caused damage to biomolecules and had cytotoxic and mutagenic effects, potentially leading to cancer. An increase in SOD and CAT was recorded in the negative control group. It should be noted that SOD and CAT are antioxidant enzymes that defend cells from free radicals produced by carcinogenic metabolism (Kalyani et al. 2017). Therefore, high SOD and CAT levels indicate a high level of oxidative stress; therefore the need for the high production of antioxidant enzymes. These results are in line with those of Ferreira and Prado (2025) who also observed lower SOD and CAT levels in the treatment groups. Lower GSH levels were recorded in the DMBA‐induced groups. Low GSH levels indicate a high level of oxidative stress. In this case, this could be as a result of the DMBA toxicity or the effect of cancer progression. GSH is one of the first lines of defense against reactive oxygen species which was reduced. Nitrites are precursors of reactive oxygen species and are also a critical risk factor of breast cancer (Mijatović et al. 2020). NO plays a role in several physiological processes including vasodilation and metabolic regulation (Andrabi et al. 2023). In this study, higher NO levels were observed in the DMBA‐induced groups compared to the normal group. However, the group receiving African walnut oil and DHA presented the least NO level compared to the other DMBA‐induced groups. These suggest that African walnut oil and DHA exert some of its anti‐cancer effects through antioxidant properties. These results correlate with the work of Zingue et al. (2024) where the group receiving “Njangsang” oil exhibited the lowest NO levels after treatment.

## 5 Conclusion

The objective of this study was to assess the effect of African walnut oil and DHA on tumor size, hematological, and oxidative stress markers of female rats with DMBA‐induced breast cancer. Data show that both docosahexaenoic acid and African walnut oil may delay tumor growth. They increase white blood cells, platelets, and mean corpuscular hemoglobin counts in the blood. DMBA significantly lowers red blood cell, hemoglobin level, and protein concentration. Docosahexaenoic acid and African walnut oil protect organs from the damages caused by oxidative stress in cancer by increasing glutathione peroxidase, catalase activities, and nitric oxide levels and increasing superoxide dismutase activity and malondialdehyde levels. Docosahexaenoic acid and African walnut oil may be used in delaying tumor progression, preserving hematological markers, and limiting oxidative stress damages in cancer patients if taken as food supplements or added to their diet.

## Author Contributions

**Fabrice Tonfack Djikeng:** conceptualization; investigation; writing – original draft; writing – review and editing; visualization; validation; methodology; software; formal analysis; supervision; resources; data curation. **Carister Nchangnwi:** conceptualization; investigation; writing – original draft; writing – review and editing; visualization; validation; methodology; software; formal analysis; resources; data curation. **Jean Paul Chedjou:** conceptualization; investigation; writing – original draft; writing – review and editing; visualization; validation; methodology; software; formal analysis; resources; supervision; data curation. **Hilaire Macaire Womeni:** conceptualization; investigation; writing – original draft; writing – review and editing; visualization; validation; methodology; software; formal analysis; supervision; data curation; resources.

## Funding

The authors have nothing to report.

## Ethics Statement

All animals were cared for and used in agreement with international standard guideline for animal use. An ethical clearance for animal handling and care was obtained from the University of Buea Institutional Animal Care and Use Committee with the reference number UB‐IACUC No. 02/2025.

## Conflicts of Interest

The authors declare no conflicts of interest.

## Data Availability

The data that support the findings of this study are available from the corresponding author upon reasonable request.
